# Silencing PRSS1 suppresses the growth and proliferation of gastric carcinoma cells via the ERK pathway

**DOI:** 10.7150/ijbs.52591

**Published:** 2021-03-01

**Authors:** Dongmei Ye, Yuxuan Li, Heliang Zhang, Zhiwei Zhou, Yujie Tang, Peng Wu, Qiang Zhao, Zhiwei Zhang

**Affiliations:** 1 Cancer Research Institute of Hengyang Medical College, University of South China; Key Laboratory of Cancer Cellular and Molecular Pathology in Hunan Province, Hunan Hengyang 421001, China.; 2 Department of Pathology, The First Affiliated Hospital of University of South China, Hunan Hengyang 421001, Hunan Province China.; 3 Department of Pathology, Third Affiliated Hospital of Nanchang University, Nanchang, Jiangxi Province 330008, China.; 4 Clinical Medicine of Hengyang Medical College, University of South China, Hengyang 421001, Hunan Province, China.; 5 Department of Critical Care Medicine, Hengyang Maternal and Child Health Hospital, Hengyang, 421001, Hunan Province, China.

**Keywords:** gastric carcinoma, proteomics, PRSS1, miR-146a-5p, ERK, PAR-2

## Abstract

**Background:** Gastric carcinoma (GC) is one of the most common malignant tumors and seriously threatens human life and health.

**Methods:** In the present study, 243 differentially expressed proteins in GC were identified using laser capture microdissection (LCM) combined with isotopically labeled quantitative proteomics technology. The expression of serine protease 1 (PRSS1) protein was analyzed by immunohistochemistry and Western blot. MTT and colony formation assays were employed to determine the effect of PRSS1 expression on the growth and proliferation of GC cells. Then, we observed the expression of miR-146a-5p in GC by qRT-PCR. A dual luciferase assay was performed to determine whether PRSS1 is a target gene of miR-146a-5p. We also explored the influence of miR-146a-5p expression on PRSS1 expression and on the growth and proliferation of GC cells. Finally, Western blotting was used to analyze the effect of PRSS1 expression on the activation of the ERK signaling pathway.

**Results:** We confirmed that PRSS1 expression was significantly increased and was positively correlated with the differentiation, tumor size and lymph node metastasis of GC. Subsequently, we found that overexpression of PRSS1 promoted the growth and proliferation of cells, whereas silencing PRSS1 expression inhibited the growth and proliferation of MGC803 cells by inhibiting activation of the ERK signaling pathway via reductions in PAR-2 activation. MiR-146a-5p targets PRSS1 and suppresses the growth and proliferation of MGC803 cells.

**Conclusions:** miR-146a-5p targets PRSS1 and suppresses the growth and proliferation of MGC803 cells. Silencing PRSS1 expression inhibits the ERK signaling pathway by reducing PAR-2 activation, resulting in suppressed growth and proliferation of MGC803 GC cells.

## Introduction

Gastric carcinoma (GC) is one of the most common malignancies, ranking fifth in global incidence among all malignancies and third in cancer-associated mortality 1,2. However, the majority of cases of GC are usually not diagnosed until an advanced stage; therefore, the outcome is often poor with a 5-year survival rate of no more than 30%, including among patients who have undergone surgery 3. It is key to improve the 5-year survival rate and quality of life of patients for the early diagnosis and management of GC. Therefore, it is worthwhile to explore methods and biomarkers for the early diagnosis of GC. However, specific and sensitive molecular markers for the early diagnosis of GC have been poorly defined.

The development of proteomics technology has provided a new way to research molecular markers for the early diagnosis of GC 4. Laser capture microdissection (LCM) technology is one of the best methods for separating cells from tissues and obtaining high-purity tissues 5. Isobaric tags for relative and absolute quantitation (iTRAQ) technology is beneficial for comparative analysis of abundance in mass spectrometry and has advantages relating to quantitative and differential expression results. The quadrupole time-of-flight tandem mass spectrometer (Q-TOF MS/MS) is suitable for the analysis of trace molecules. This machine is significantly better than traditional techniques for not only its resolution, accuracy and sensitivity but also its ability to determinate proteins with very low molecular weight, extreme pH and low abundance 6.

The PRSS1 gene is located on human chromosome 7 and encodes 247 amino acids, comprising cationic trypsin 1. Recent studies have shown that the expression of PRSS1 protein is significantly increased in pancreatic cancer, colorectal cancer and cervical cancer and may be involved in the pathological process of tumor progression 7-9. MicroRNAs (miRNAs) are a type of noncoding RNA widely found in eukaryotes. They mainly recognize the 3′-UTR of their target mRNA to either degrade the mRNA or inhibit its translation 10. MiR-146a-5p is dysregulated in a variety of cancers and plays an important role in the development of cancer 11,12. Previous studies reported that miR-146a-5p is dysregulated in GC 13; however, the signaling mechanisms need further research. Protease-activated receptor-2 (PAR-2) is a seven-transmembrane G protein-coupled receptor consisting of 397 amino acid residues that is expressed in a variety of tumor cells. It has been reported that various proteases can exert hydrolytic activity and activate PAR-2 14, which consequently affects tumor proliferation, metastasis and angiogenesis through the ERK pathway 15. However, it remains unclear whether PAR-2 activation affects the proliferation of gastric cancer cells.

In this study, we identified differentially expressed proteins in LCM-purified gastric mucosal epithelial cancerous tissues using iTRAQ labeling and 2D LCMS/MS and selectively verified a subset of the differentially expressed proteins with consistent results. Among them, PRSS1 protein was significantly overexpressed, which was positively correlated with the differentiation, tumor size and lymph node metastasis of GC. Subsequently, PRSS1 is a target gene of miR-146a-5p. Finally, we found that knockdown of PRSS1 expression inhibited activation of the ERK signaling pathway by reducing PAR-2 activation, resulting in suppressed proliferation of GC MGC803 cells.

## Materials and methods

### Patients

We collected 20 matched pairs of fresh normal gastric mucosa (NGM), atypical hyperplasia (AH), poorly differentiated gastric adenocarcinoma (GPDAC) and lymph node metastasis adenocarcinoma (LMGAC) tissues for LMC (Supplementary Table 1). In addition, 81 pairs of GC and adjacent normal formalin-fixed paraffin-embedded tissues were collected for immunohistochemical (IHC) staining with a PRSS1 antibody. Tissue chips made from 141 GC specimens were used for IHC staining with a PAR-2 antibody. All samples were diagnosed in the Department of Pathology of the First Affiliated Hospital of University of South China. All samples were obtained from patients with GC with approval of the medical ethics committee.

### Laser capture microdissection (LCM)

Tissue samples were removed from liquid nitrogen and sliced into 8-µm-thick frozen sections using a cryostat device carrier (Leica Biosystems GmbH, Wetzlar, NJ). Frozen tissue sections were stained with methyl green and placed on an LCM (Leica LMD6, Leica Microsystems GmbH). The target tissues were outlined on the display and automatically cut from the slice with the laser. We collected the target tissues in a tube containing 2-3 µl of protease inhibitor (Roche Diagnostics, Basel, Switzerland) and stored them at -80 °C.

### Protein extraction and isobaric tags for relative and absolute quantitation (iTRAQ) labeling

Total protein from LMC-treated tissues was extracted using lysis buffer (CW2333S, Cwbiotech, Beijing, China) following the manufacturer's protocol. The total proteins from NGM, AH, GPDAC and LMGAC tissue samples were labeled with different iTRAQ reagents (Thermo Fisher Scientific, Waltham, MA, USA) according to the manufacturer's instructions. Four labeled lysates were mixed and lyophilized before they were desalted. Then, the samples were dissolved in deionized water containing 0.1% formic acid (FA, Tedia Company, Fairfield, OH, USA) and eluted three times with Sep-Pak C18 1 cc Vac cartridges (Waters Corporation, Milford, MA, USA). The cleaning solution was collected and lyophilized to obtain the final samples.

### 2D LC-ESI-MS/MS analysis and identification of differentially expressed proteins

The samples marked with iTRAQ were dissolved in cation-exchange (SCX) buffer for SCX separation. The four samples containing mesenchymal proteins from NGM, AH, GPDAC and LMGAC tissues were mixed and loaded into a polysulfoethyl column and segregated by an LC-20AD high-performance liquid chromatography (HPLC) system (Shimadzu Corporation, Kyoto, Japan) following the manufacturer's instructions. Next, the products were concentrated by vacuum centrifugation for reverse-phase HPLC-mass spectrometry (MS) analysis. The samples were dissolved in 50 µl of 5% ACN containing 0.1% FA and loaded into a Zorbax 300SB-C18 column (Agilent Technologies, Santa Clara, CA, USA) according to the manufacturer's instructions. The data were analyzed using QSTAR-XL (Applied Biosystems, Thermo Fisher Scientific) and tandem MS (MS/MS). Finally, the IPI human database (version 3.45, URL: http://www.ebi.ac.uk/IPI) was searched for protein information, the confidence level was set as >95%, and the ion peak areas of m/z 114 and 118 were integrated.

### Cell line and cell culture

GC cell lines (SGC7901, BGC823, MGC803) and the immortalized gastric epithelial cell line GES-1 were obtained from Cancer Research Institute of the University of South China for the present study. HEK293T cells were obtained from the ATCC (American Type Culture Collection, Manassas, VA). Cells were cultured in either RPMI 1640 (HyClone) medium containing 10% fetal bovine serum (HyClone) or serum-free keratinocyte medium (K-SFM, Gibco, Thermo Fisher Scientific, Waltham, MA, USA) in a humidified 5% (v/v) CO2 atmosphere at 37 °C.

### Plasmids

The PRSS1 interference vector pcDNA6.2^TM^-GW/EmGFP-miR-PRSS1 (miR-PRSS1), high expression vector pcDNA3.1-PRSS1 and corresponding negative control (NC) plasmids (pcDNA6.2^TM^-GW/EmGFP-miR (PRSS1-NC) and pcDNA3.1-NC, respectively) were purchased from Shanghai Invitrogen. PRSS1-WT and PRSS1-MUT vectors were constructed by Fenghui Biological. The sequences of all the vectors were confirmed by Sangon Biotech.

### Transfection

Transfections were performed with Lipofectamine 2000 Reagent (Invitrogen) according to the manufacturer's protocol. MGC803 cells were seeded in a 6-well plate at 1×10^5^/well. We transfected the interference vector pcDNA6.2™-GW/EmGFP-miRi-PRSS1 and the pcDNA6.2™-GW/EmGFP-miRi NC vector into MGC803 cells to construct MGC803/miRi-PRSS1 (low expression of PRSS1) and MGC803/miRi NC cells, respectively. As the same time, GES-1 cells were seeded in a 6-well plate at 1×105 cells/well. We transfected pcDNA3.1-PRSS1 and pcDNA3.1 NC into GES-1 cells to construct PRSS1-overexpressing and NC cells, respectively. The transfection efficiency was observed by fluorescence microscopy and Western blot analysis.

### Western-blot analysis

Total cellular proteins were separated via SDS-PAGE through 12% gels and transferred to polyvinylidene difluoride membranes. The membranes were blocked with QuickBlock™Blocking Buffer for Western blot (P0252, Beyotime, Shanghai, China) for 10 minutes and incubated with primary antibodies targeting PRSS1 (ab200996, 1:1000, Abcam, UK), HSP90α/β (1:500, Santa Cruz Biotechnology, USA), TGM2 (1:1000, CST, USA), SerpinA3 (1:1000, Santa Cruz Biotechnology, USA), P180 (1:500, Aibixin, Shanghai, China), PCNA (ab137867, 1:1000, Wanleibio, Shanghai, China), ERK1/ERK2 (ab184699, 1:5000, Abcam, UK), ERK1/ERK2 (ab76299, 1:5000, Abcam, UK), or β-actin (1:1000, Sigma-Aldrich; Merck KGaA, Darmstadt, Germany) at 4 °C overnight. Fluorescent secondary antibody (1:5000; Santa Cruz Biotechnology) was then added to the membranes for 2 hours. The protein bands were detected by a gel imaging analysis system (ODYSSEY Sa, LI-COR, USA).

### Immunohistochemical staining

Samples were fixed with 10% formalin, and 4-µm-thick paraffin sections were baked in a 58 °C incubator for 2 hours. The immunohistochemical staining procedure was performed following the S-P kit instructions (Maixin, Fujian, China). The cells were incubated with antibodies targeting PRSS1 (ab200996, 1:1000, Abcam, UK), PCNA (ab137867, 1:500, Wanleibio, Shanghai, China), and PAR-2 (ab1809, 1:100, Abcam, UK) overnight at 4 °C, which were then detected by adding DAB. Finally, the cells were observed under a microscope (BX53, Olympus, Japan).

### Quantitative reverse transcription polymerase chain reaction (qRT-PCR)

Total RNA from cells was isolated using TRIzol reagent (Invitrogen, USA) according to the manufacturer's protocol. We synthesized complementary DNA using the All-in-One™ FIST-Strand cDNA miRNA Synthesis Kit (QP013, GeneCopoeia, USA), and RT-PCR was performed using All-in-One™ miRNA qRT-PCR (QP015, GeneCopoeia, Rockville, MD, USA) and BeyoFast^TM^ SYBR Green qRT-PCR Detection kits (Beyotime Biotechnology, Shanghai, China). Primers targeting miR-146a-5p (HmiRQP0196), U6 (HmiRQP9001) and PRSS1 (HQP015083) were purchased from GeneCopoeia. The primers for the GAPDH gene were synthesized by Sangon Biotech as follows: 5′-AGGTCGGTGTGAACGGATTTG-3′ (forward) and 5′-GGGGTCGTTGATGGCAACA-3′ (reverse).

### MTT assay

Cells were seeded into 96-well plates at 2×10^3^ cells/well. The number of viable cells was detected by the MTT method according to the manufacturer's instructions (Molecular Probes, Eugene, OR, USA) every 24 hours in triplicate. The OD values were measured at 570 nm with a microplate reader (17260, BIO-RAD, USA).

### Colony formation assay

The cells were suspended before they were seeded in a six-well plate at 1×10^3^ cells/well at 37 °C for 2 weeks. The colonies were fixed with methanol for 25 min and then stained with 0.4% crystal violet for 30 minutes at room temperature (E607309, BBI, Shanghai, China). The number of cell colonies was counted under a microscope (TS100, Nikon, Japan) and analyzed.

### Soft agar clone formation assay

Cells were seeded into semisolid agar K-SFM medium [base layer, 0.6% (w/v); upper layer, 0.3% (w/v)] at a density of 5×10^4^ cells/well in 6-well plates. After 2-week incubation at 37 °C with 5% (v/v) CO2, the number and size of colonies (≥50 cells as one colony) were observed and analyzed.

### Immunocytochemical staining

Cell slides were prepared and fixed with 4% paraformaldehyde for 30 min. The immunocytochemical staining procedure was the same as that used for IHC according to the S-P kit instructions (Maixin, Fujian, China). The cells were incubated with antibodies targeting proliferating cell nuclear antigen (PCNA) (ab137867, 1:500, Wanleibio, Shanghai, China) and PAR-2 (ab1809, 1:100, Abcam, UK) overnight at 4 °C and stained by adding DAB. Finally, the cells were observed under a microscope (BX53, Olympus, Japan).

### Luciferase reporter assay

PRSS1-WT and PRSS1-MUT are vectors containing the recombinant wild-type and mutant form of PRSS1, respectively. All vectors were confirmed by sequencing (Sangon Biotech). HEK 293T cells were seeded into 24-well plates at a density of 5×10^4^ cells/well and cotransfected with the PRSS1-WT or PRSS1-MUT plasmids and the miR-146a-5p mimics or NC using LipoFilter transfection reagent (HB-TRLF-1000, Hanheng, Shanghai, China). Luciferase activity was measured by a dual-luciferase reporter assay system (Lux-T020, BLT, Guangzhou, China) after 48 hours of transfection following the manufacturer's instructions. Relative luciferase activity was expressed as the fold-change after normalization to Renilla luciferase activity. Recombinant expression vectors were confirmed by sequencing (Sangon Biotech).

### Statistical analysis

Statistical analyses were performed using GraphPad Prism 8 software. Data are expressed as the means±standard deviation (M±SD). The results of the immunohistochemical staining were analyzed by the Kruskal-Wallis H test, and Fisher's exact probability test was used for pairwise comparisons. One-way ANOVA was used to compare the means of samples between groups. The MTT experiment results were statistically analyzed using two-way ANOVA. Student's t-test was performed when the variance between groups was similar, and the correlation test used was Spearman's correlation analysis. *P<0.05, **P<0.01, and ***P<0.001 were considered to indicate a statistically significant difference.

## Results

### Identification of differentially expressed proteins during human gastric mucosal carcinogenesis by Proteomics

**Figure 1:**
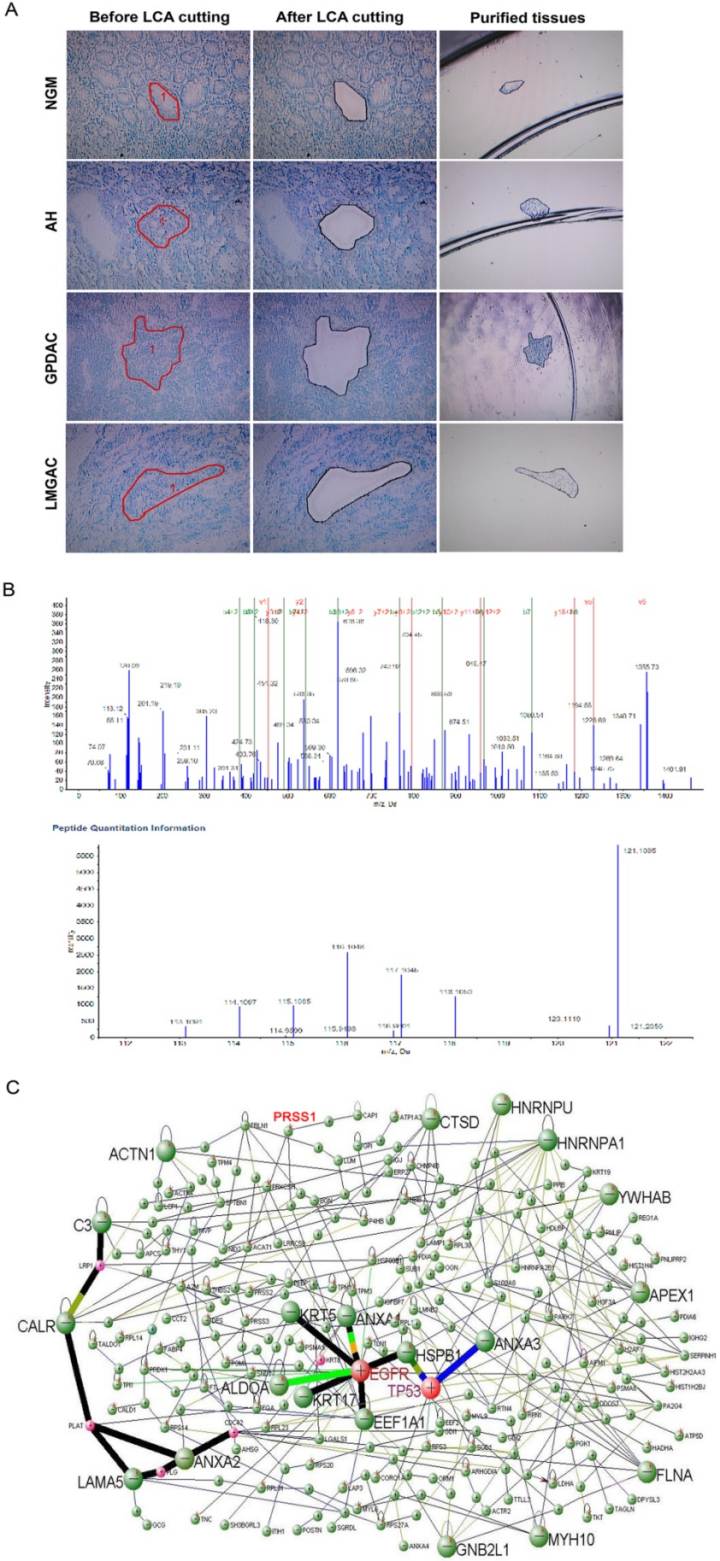
** Identification of differentially expressed proteins during human gastric mucosal carcinogenesis by proteomics analysis.** (A) LCM of purified NGM, AH, GPDAC and LMGAC tissues. (B) MS/MS maps for identifying peptides of PRSS1 and relative quantitative information of PRSS1 expression in four different tissues. (C) A regulatory network of gastric mucosa carcinogenesis-related proteins. LCM, laser capture microdissection; NGM, normal gastric mucosa; AH, atypical hyperplasia; GPDAC, poorly differentiated gastric adenocarcinoma; LMGAC, lymph node metastasis adenocarcinoma.

To explore differentially expressed proteins related to gastric mucosa carcinogenesis, we used LCM technology to extract proteins from 20 normal gastric mucosa (NGM), atypical hyperplasia (AH), poorly differentiated gastric adenocarcinoma (GPDAC) and lymph node metastasis adenocarcinoma (LMGAC) tissues (Figure 1A). A total of 243 differentially expressed proteins were identified by iTRAQ labeling, two-dimensional liquid chromatography separation and Q-TOF MS/MS mass spectrometry (the Life Science Research Center of Fudan University). The MS/MS map of PRSS1 peptides is shown in Figure 1B. There were significant differences in the levels of the PRSS1, TGM2, SerpinA3, P180 and HSP90α/β proteins, and their expression gradually increased in NGM, AH, GPDAC and LMGAC tissues (Table 1). We constructed a regulatory network of gastric mucosa carcinogenesis-related proteins via DAVID Online Bioinformatics Analysis Software (http://david.abcc.ncifcrf.gov) (Figure 1C).

### Expression and clinical significance of PRSS1 in GC

**Figure 2:**
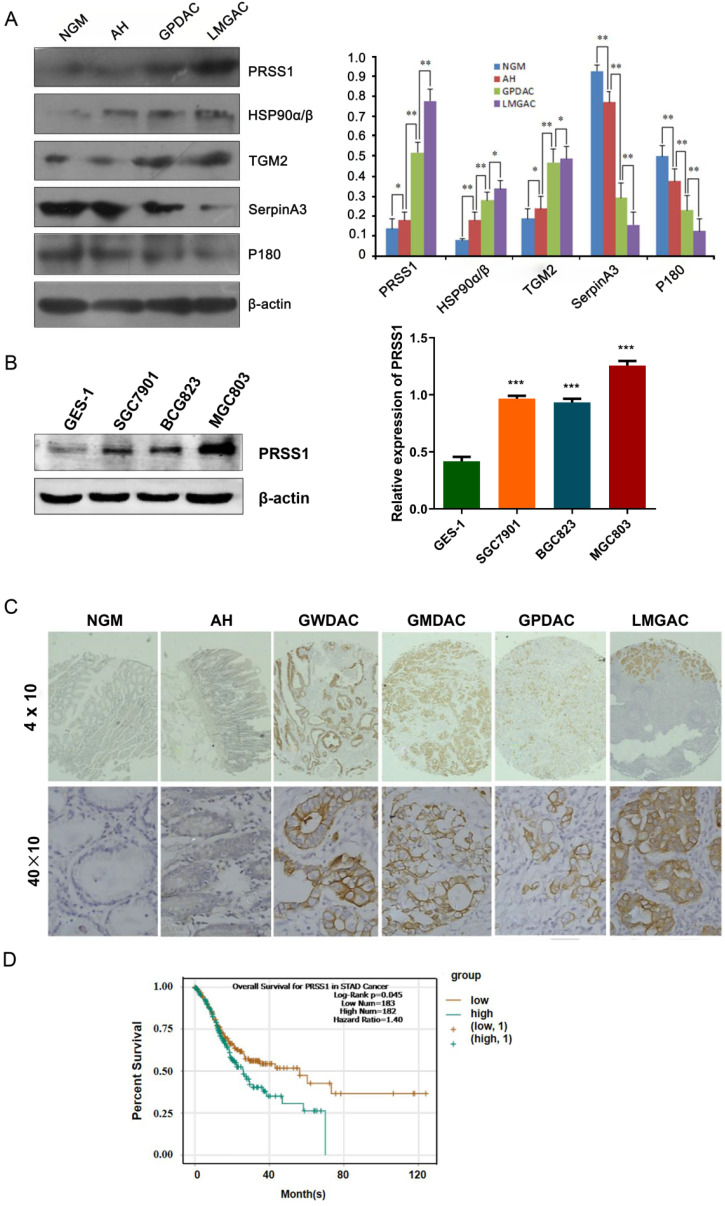
** Expression and clinical significance of PRSS1 in GC.** (A) Western blotting was performed to detect the expression of PRSS1, HSP90α/β, TGM2, SerpinA3 and P180 in purified NGM, AH, GPDAC and LMGAC tissues. (B) PRSS1 was highly expressed in GC cells. (C) Immunohistochemical staining analysis indicated that PRSS1 was highly expressed in tissues. (D) Patients with high expression of PRSS1 had poor overall survival. GC, gastric cancer; SD, standard deviation. Data are shown as the means ± SD. *P<0.05, **P<0.01, ***P<0.001.

To further confirm the quantitative proteomics results, we detected the expression levels of some of the differentially expressed proteins in 20 NGM, AH, GPDAC, and LMGAC tissues by Western blot. The results of the Western blot analysis were consistent with those of the quantitative proteomics analysis (Figure 2A). Having demonstrated that, we evaluated the expression and clinical significance of PRSS1 in GC. Western blot and IHC staining analyses indicated that PRSS1 was highly expressed in GC cells and tissues (Figure 2B, 2C and Table 2). Subsequently, we explored the correlation between PRSS1 expression and the clinicopathological status of GC patients. According to statistical analysis, PRSS1 overexpression was positively correlated with the differentiation, tumor size and lymph node metastasis of GC (Table 3). Then, we predicted the clinical significance of PRSS1 in patient prognosis. As shown in Figure 2D, there was poor overall survival (OS) of patients with high expression of PRSS1. Therefore, PRSS1 may be a potential marker for the early diagnosis and prognosis of GC.

### Effect of PRSS1 expression on the growth and proliferation of GC cells

**Figure 3:**
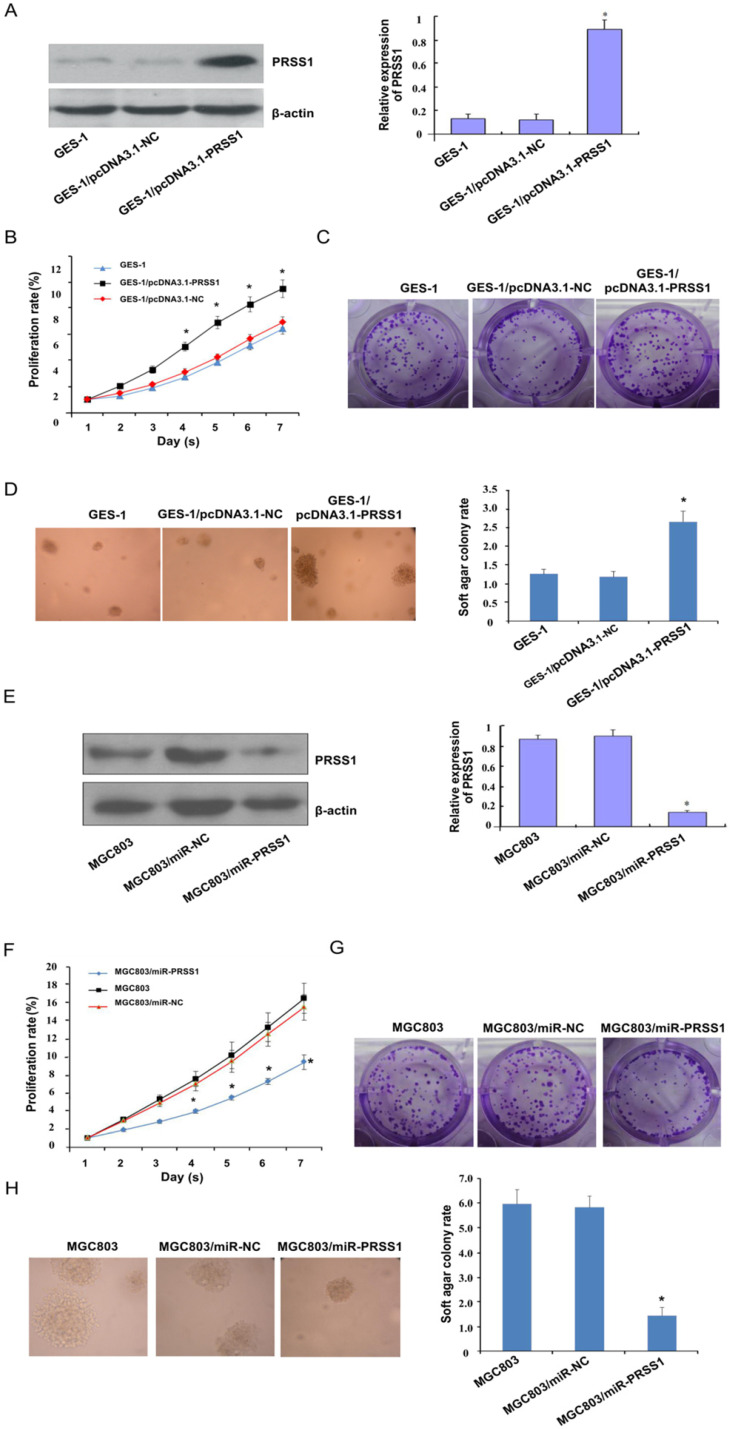
** Effect of PRSS1 expression on the growth and proliferation of GC cells.** (A) The expression level of PRSS1 protein was significantly increased in GES-1/pcDNA3.1-PRSS1 cells. (B-D) Overexpression of PRSS1 significantly increased the growth and proliferation of GES-1 cells. (E) The expression level of PRSS1 protein in MGC803/miR-PRSS1 cells was significantly decreased. (F-G) Knockdown of PRSS1 expression significantly decreased the growth and proliferation of MGC803 cells. GES-1, immortalized gastric mucosal epithelial cells; GC, gastric cancer. Data are shown as the means ± SD, *P<0.05.

Subsequently, we investigated the effect of PRSS1 expression on the growth and proliferation of GC cells. A GES-1 cell line with ectopic expression of PRSS1 (GES-1/pcDNA3.1-PRSS1) and an MGC803 cell line with knockdown of PRSS1 expression (MGC803/miR-PRSS1) were established (Figure 3A and 3E). We performed MTT, colony formation and soft agar colony formation experiments to examine the changes in the proliferation of GES-1/pcDNA3.1-PRSS1 cells and GC MGC803/miR-PRSS1 cells. The MTT assays showed that overexpression of PRSS1 significantly increased the growth of GES-1 cells, whereas downregulation of PRSS1 significantly inhibited the growth of MGC803 cells (Figure 3B and 3F). In the colony formation and soft agar assays, the colony-forming ability of GES-1 cells was significantly increased after upregulation of PRSS1 expression; in contrast, the colony-forming ability of MGC803 cells was significantly reduced after downregulation of PRSS1 expression (Figure 3C-D and 3G-H). The results indicated that increased expression of PRSS1 promoted cell growth and proliferation, and silencing PRSS1 expression inhibited the growth and proliferation of MGC803 cells.

### MiR-146a-5p targets PRSS1 and inhibits MGC803 cell growth and proliferation

**Figure 4:**
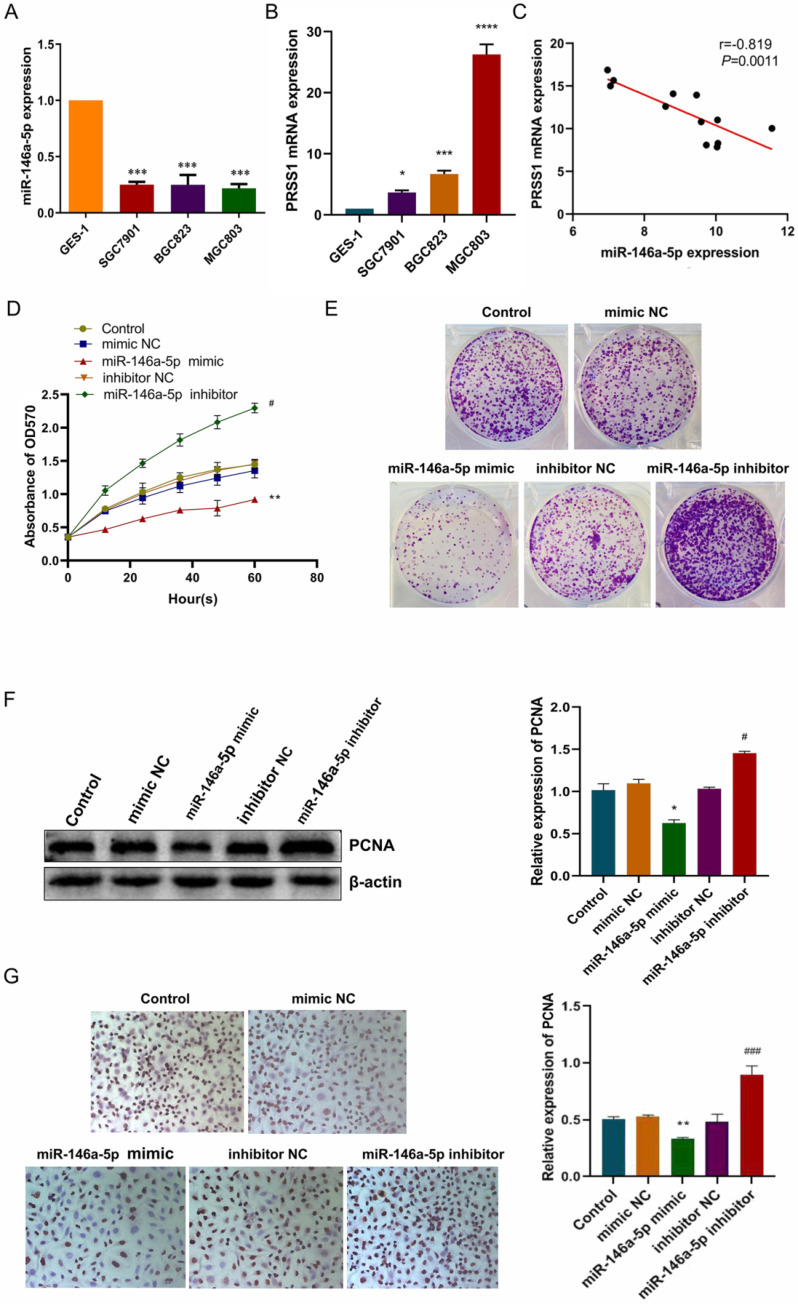
** MiR-146a-5p targets PRSS1 and inhibits MGC803 cell growth and proliferation.** (A) MiR-146a-5p expression was significantly decreased in GC cells. (B) PRSS1 mRNA expression was significantly increased in GC cells. (C) The expression level of miR-146a-5p was negatively correlated with PRSS1 mRNA expression as indicated by qRT-PCR. (D-E) The effects of MGC803 cells transfected with miR-146a-5p mimic or miR-146a-5p inhibitor on growth and colony-forming ability were determined by the MTT and colony forming assays. (F-G) Western blot and ICC analyses showed that miR-146a-5p mimic reduced PCNA protein expression while miR-146a-5p inhibitor increased PCNA expression. Data are shown as the means ± SD. ^*^P<0.05, ^**^P<0.01, ^***^P<0.001; ^#^*P<*0.05, ^###^*P<*0.001.

Next, we used miwalk and miRanda bioinformatics software to find whether miR-146a-5p has a potential binding site with PRSS1. We confirmed that miR-146a-5p expression was significantly decreased in GC cells (Figure 4A) while PRSS1 mRNA was significantly increased in the same cells (Figure 4B). The expression level of miR-146a-5p was negatively correlated with PRSS1 mRNA expression as measured by qRT-PCR (Figure 4C). To investigate whether miR-146a-5p affects the growth and proliferation of MGC803 cells, we transfected miR-146a-5p mimic, mimic NC, inhibitor, and inhibitor NC into MGC803 cells and found that the growth and colony-forming ability of MGC803 cells were significantly reduced after miR-146a-5p expression was upregulated. Conversely, the growth and colony-forming ability of MGC803 cells were significantly increased after miR-146a-5p expression was downregulated (Figure 4D-E). At the same time, Western blot analysis showed that miR-146a-5p mimic reduced the protein expression of proliferating cell nuclear antigen (PCNA), while the miR-146a-5p inhibitor increased PCNA expression (Figure 4F). The immunocytochemistry (ICC) results were consistent with the Western blot results (Figure 4G).

### PRSS1 mRNA is a direct target of miR-146a-5p

**Figure 5:**
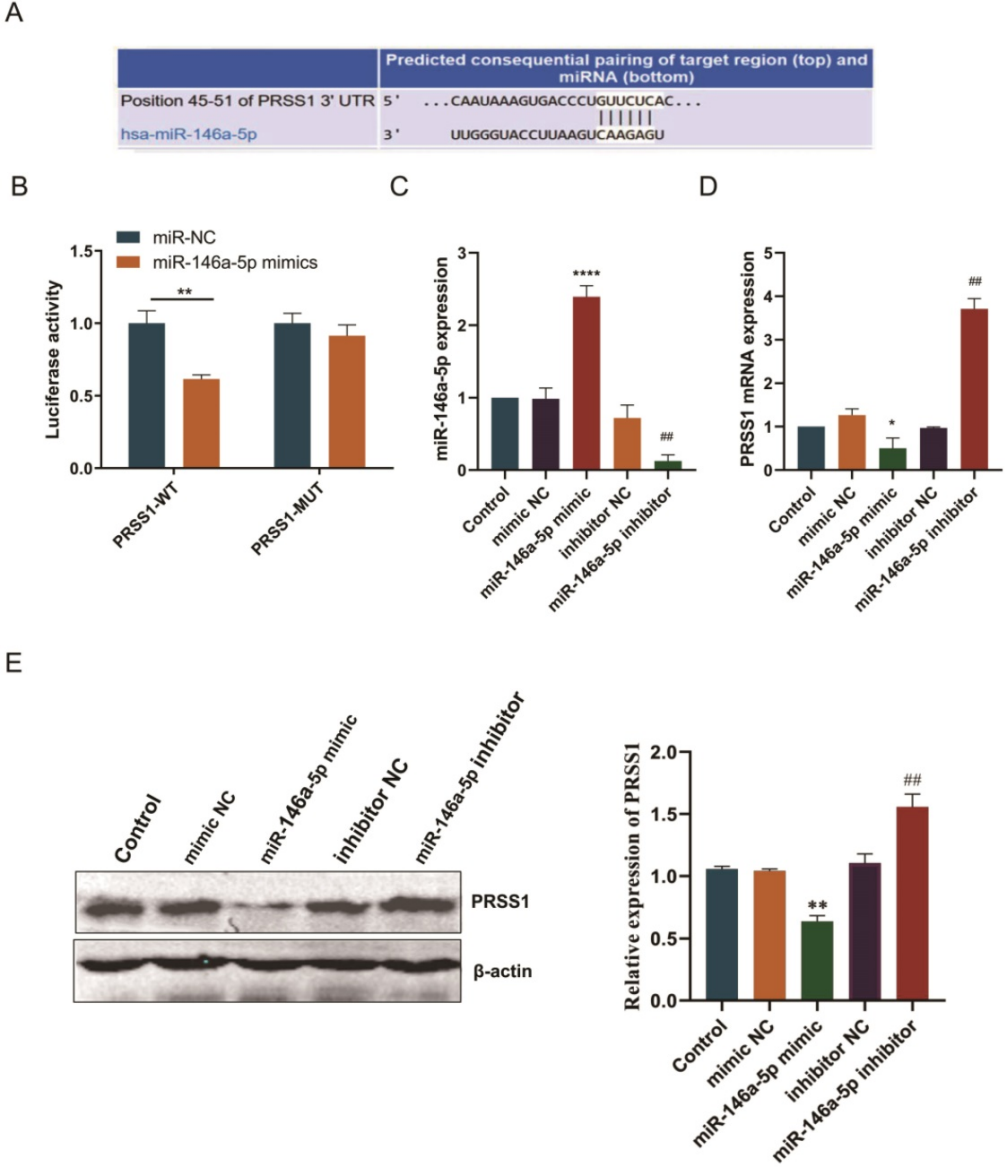
** PRSS1 is a direct target gene of miR-146a-5p.** (A) The TargetScan database was used to predict the binding site of miR-146a-5p to PRSS1. (B) Luciferase assay of HEK 293T cells cotransfected with miR-146a-5p mimic, miR-146a-5p mimic NC and a luciferase reporter containing the wild-type 3′UTR of PRSS1 (PRSS1-3′UTR-wt) or mutated miR-146a-5p binding sites on the 3′UTR (PRSS1-3′UTR-mut). (C-D) qRT-PCR was performed to detect the expression changes of miR-146a-5p and PRSS1 mRNA in MGC803 cells transfected with miR-146a-5p mimic and miR-146a-5p inhibitor, respectively. (E) The effect of miR-146a-5p mimic or miR-146a-5p inhibitor on PRSS1 protein expression in MGC803 cells was determined by Western blot. Data are shown as the means ± SD. ^*^P<0.05, ^**^P<0.01, ^***^P<0.001; ^#^*P<*0.05, ^##^*P<*0.01.

Subsequently, we predicted the binding site of miR-146a-5p to PRSS1 mRNA via the TargetScan database (Figure 5A). To confirm this finding, we performed a luciferase reporter experiment to verify whether miR-146a-5p directly targets PRSS1 mRNA. First, we constructed luciferase reporters containing the wild-type and mutant sequences of the PRSS1 3′UTR. We cotransfected miR-146a-5p mimic or NC with each luciferase reporter gene into HEK 293T cells and measured luciferase activity. The results indicated a significant reduction in luciferase activity when wild-type PRSS1 (PRSS1-WT) was cotransfected with miR-146a-5p. However, there was a significant increase in luciferase activity when mutant PRSS1 (PRSS1-MUT) was cotransfected with miR-146a-5p (Figure 5B). In addition, we further confirmed the results of the luciferase assays by qRT-PCR and Western blot analysis. Our data demonstrated that the miR-146a-5p mimic significantly downregulated the expression of PRSS1 mRNA and protein, while the miR-146a-5p inhibitor upregulated the expression of PRSS1 mRNA and protein (Figure 5C-E). Therefore, PRSS1 is a direct target gene of miR-146a-5p, and miR-146a-5p inhibits the growth and proliferation of GC by downregulating the expression of PRSS1.

### PRSS1 affects cell proliferation via the PAR-2-activated ERK signaling pathway

**Figure 6:**
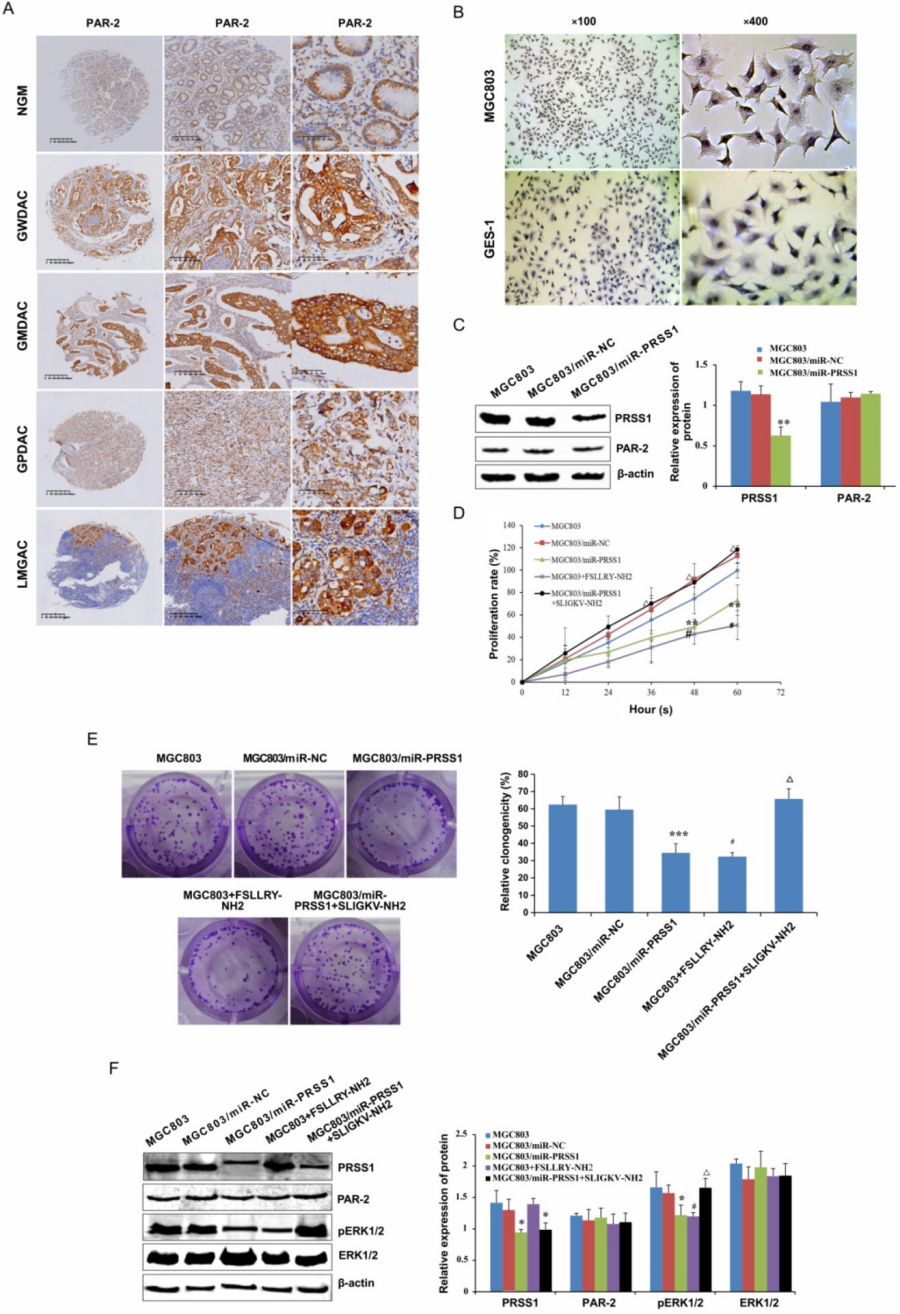
** PRSS1 affects cell proliferation via the PAR-2-activated ERK signaling pathway.** (A) The expression of PAR2 protein was analyzed in GC tissues by immunohistochemistry analysis. (B) PAR-2 was highly expressed in MGC803 GC cells compared to GES-1 cells. (C) The effect of PRSS1 knockdown on PAR-2 protein expression in MGC803 cells. (D-E) The effect of FSLLRY-NH2 (PAR-2 inhibitor) and SLIGKV-NH2 (PAR-2 agonist) on the growth and proliferation of MGC803 GC cells was determined by MTT and colony formation assays. (F) Western blotting was performed to detect the expression levels of phosphorylated ERK1/2, PRSS1 and PAR2 in MGC803/miR-PRSS1 cells, MGC803 cells treated with FSLLRY-NH2 (PAR-2 inhibitor), and MGC803/miR-PRSS1 cells treated with SLIGKV-NH2 (PAR-2 agonist). Data are shown as the means ± SD,^ *^P<0.05, ^**^P<0.01, ^***^P<0.001; ^#^*P<*0.05, ^##^*P<*0.01; ^Δ^*P<*0.05.

In searching for the molecular mechanism downstream of PRSS1 that affects GC MGC803 cell proliferation, we found that PAR-2 was highly expressed in well-differentiated, moderately differentiated and poorly differentiated gastric adenocarcinoma and lymph node metastatic cancer tissues compared with normal gastric mucosa (Figure 6A, Table 4) as measured by IHC. The expression of PAR-2 in GC tissues was associated with TNM stage and lymph node metastasis (Table 5). Moreover, compared with GES-1 cells, MGC803 GC cells showed high expression of PAR-2 (Figure 6B). We transfected the miR-PRSS1 interference plasmid into MGC803 cells to silence the expression of PRSS1, but there was no significant difference in the expression of PAR-2 (Figure 6C). Then, we treated MGC803 cells with FSLLRY-NH2 (PAR-2 inhibitor) and SLIGKV-NH2 (PAR-2 agonist) and observed cell growth and proliferation as well as measured the levels of phosphorylated ERK1/2. The results indicated that cell growth and proliferation and the levels of phosphorylated ERK1/2 in MGC803/miR-PRSS1 cells were significantly reduced compared those in with MGC803 and MGC803/miR-NC cells (Figure 6D-F). Additionally, cell growth, proliferative ability and levels of phosphorylated ERK1/2 were reduced in MGC803 cells treated with FSLLRY-NH2 (Figure 6D-F). Interestingly, when SLIGKV-NH2 was added to MGC803/miR-PRSS1 cells, cell growth, proliferative ability and phosphorylated ERK1/2 levels were restored (Figure 6D-F). However, we found that the changes in PRSS1 expression and the addition of either FSLLRY-NH2 or SLIGKV-NH2 did not affect the expression level of PAR-2 protein (Figure 6F). The results suggest that knockdown of PRSS1 expression inhibits ERK signaling pathway activation by reducing PAR-2 activation, resulting in weakened growth and proliferation of MGC803 cells.

## Discussion

Improving the 5-year survival rate and quality of life of patients is key for the early diagnosis and management of GC. There is an urgent need to identify of specific and sensitive molecular markers for the early diagnosis of GC. The development of proteomics technology has provided a new way to research molecular markers for the early diagnosis of GC 4. In this study, we identified differential PRSS1 proteins in gastric mucosal epithelial cancerous tissue via iTRAQ labeling and 2D LCMS/MS.

A previous report showed that PRSS1 may be an oncogene for pancreatic tumors 16. Recent studies have shown that the PRSS1 protein is important in the development of pancreatic, colorectal and cervical cancer 7-9. However, whether and how the PRSS1 protein is involved in the progression of GC is unknown. In the present study, we confirmed that PRSS1 was highly expressed in GC, which was positively correlated with tumor differentiation, tumor size, TNM stage and lymph node metastasis of GC and was associated with poor prognosis. Cell abnormalities and unchecked proliferation are hallmark features of malignant tumors 17. Moreover, we found that PRSS1 overexpression promoted cell growth and proliferation, while the opposite result was obtained when PRSS1 expression was knocked down. Therefore, PRSS1 may play an oncogenic role in GC and be involved in the process of GC. However, the underlying mechanism by which PRSS1 affects GC is poorly understood.

MicroRNAs are small noncoding RNAs that regulate the expression of target genes through posttranscriptional modification and are involved in tumor progression 18. Previous studies have reported that miR-146a-5p is involved in the occurrence and development of multiple tumors 11,19. Other reports have demonstrated that miR-146a-5p expression levels are significantly reduced in GC, which inhibits the proliferation, migration and invasion of GC 20,21. However, some studies have shown that miR-146a-5p is highly expressed in GC and promotes the progression of GC 22,23. In our study, we confirmed that miR-146a-5p expression is significantly reduced in GC, and increasing the expression of miR-146a-5p inhibits the growth and proliferation of GC cells. In contrast, we observed a decrease in cell growth and proliferation in MGC803 cells lacking miR-146a-5p expression. In addition, PRSS1 is a direct target gene of miR-146a-5p. Our data indicated that miR-146a-5p targets PRSS1 and inhibits the growth and proliferation of MGC803 cells.

PAR-2, a member of the family of protease-activated receptors widely distributed on the surface of human cells, is mainly activated by proteases 24. According to the PAR-2 activation mechanism, there are multiple modulators of PAR-2 activity that are commonly used: the PAR-2 agonist SLIGKV-NH2 and the PAR-2 inhibitor FSLLRY-NH2 25. These regulators are powerful tools for PAR-2-related research. PAR-2 is highly expressed in GC and may be associated with GC proliferation and angiogenesis 26,27. Chen reported that PAR-2 activation plays a key role in maintaining tumor cell proliferation 28. In our cell proliferation assays, both knockdown of PRSS1 expression and inhibition of PAR-2 activity effectively inhibited MGC803 cell growth and proliferation; however, the growth and proliferative ability of MGC803/miR-PRSS1 cells treated with the PAR-2 agonist SLIGKV-NH2 was completely restored. This suggested that low expression of PRSS1 leads to a decrease in the PAR-2 activation level in MGC803 cells, indicating that PRSS1 plays an important role in maintaining cell growth and proliferation in a PAR-2-dependent manner.

PRSS1, a T cell receptor protein, is an important molecule for tumor signal transduction and activity regulation. PRSS1 activates receptors or ligands on the cell surface and is involved in cell growth, proliferation, differentiation and migration through the MAPK/ERK signaling pathway 29-31. The ERK signaling pathway is a classic pathway of the mitogen-activated protein kinase (MAPK) family 32, which is mainly responsible for transmitting extracellular signals to the nucleus and regulating cell proliferation, differentiation, metastasis and survival 33. Activated PAR-2 is involved in ERK signaling and mediated tumor cell proliferation 34. However, the function of the ERK signaling pathway in GC needs further study. Phosphorylated ERK1/2 is an important marker of ERK signaling pathway activation 35. In the present study, we confirmed that knockdown of PRSS1 expression significantly inhibited the activation of the ERK signaling pathway, and the inhibitory effect was comparable to that of MGC803 cells treated with FSLLRY-NH2. Interestingly, when MGC803/miR-PRSS1 cells were incubated with SLIGKV-NH2, the level of phosphorylated ERK1/2 was completely restored. Our data indicated that knockdown of PRSS1 expression prevents the activation of the ERK signaling pathway by reducing PAR-2 activation in MGC803 cells. This is consistent with previous reports that PAR-2 activation may affect ERK signaling pathway activation 36.

Together, our data demonstrate that PRSS1 protein expression was significantly increased in GC and was positively correlated with differentiation, tumor size, and lymph node metastasis. In addition, PRSS1 is a target gene of miR-146a-5p. MiR-146a-5p targets PRSS1 and suppresses the growth and proliferation of MGC803 cells. Silencing PRSS1 expression inhibits ERK signaling pathway activation by reducing PAR-2 activation, resulting in suppressed growth and proliferation of MGC803 cells. Further research and attention on PRSS1 will help us better understand the occurrence and progression of GC and provide novel evidence for the identification of biomarkers or potential targets for the early treatment of GC.

## Supplementary Material

Supplementary tables.

## Figures and Tables

**Table 1:** Differentially expressed proteins during human gastric mucosa epithelial carcinogenesis

No.	Accession#	Protein Name	AH *vs*. NGM	GPDAC *vs*. NGM	LMGAC *vs*. NGM	GPDAC *vs*. AH	LMGAC *vs*. AH	LMGAC *vs*. GPDAC
1	IPI00465084.6	Desmin	↓0.098	↓0.024		↓0.023	↓0.123	↓0.016
2	IPI00027720.1	PnLIP Pancreatic triacylglycerol lipase precursor	↓0.229	↓0.024	↓0.177	↓0.107		↓0.138
3	IPI00020987.1	PRELP Prolargin precursor	↓0.153	↓0.025	↓0.175	↓0.163		↓0.142
4	IPI00009826.2	Carboxypeptidase B precursor	↓0.087	↓0.027	↓0.063		↓0.253	↓0.425
5	IPI00025476.1	AMY1B Pancreatic alpha-amylase precursor	↓0.449	↓0.028	↓0.115	↓0.061	↓0.256	↓0.240
6	IPI00878546.1	Protein disulfide isomerase family A, member 2	↓0.065	↓0.030	↓0.093	↓0.461		↓0.322
7	IPI00009823.3	Carboxypeptidase A1 precursor	↓0.163	↓0.053		↓0.328	↓0.411	
8	IPI00021885.1	FGA Isoform 1 of Fibrinogen alpha chain precursor	↓0.316	↓0.069	↓0.131	↓0.219	↓0.413	↓0.530
9	IPI00005924.4	PnLIPRP2 pancreatic lipase-related protein 2	↓0.051	↓0.084	↓0.136	↓0.608		↓0.619
10	IPI00010796.1	P4HB Protein disulfide-isomerase precursor	↓0.175	↓0.089		↓0.506	↓0.637	
...	...	...	...	...	...	...	...	...
24	IPI00218320.4	TPM3 Isoform 3 of Tropomyosin alpha-3 chain		↓0.205		↓0.608	↑1.047	↓0.581
25	IPI00026271.5	Ribosomal protein S14	↓0.174	↓0.223	↓0.555			↓0.402
26	IPI00219217.3	L-lactate dehydrogenase B chain	↓0.119	↓0.227		↓0.134	↓0.421	↓0.319
27	IPI00515087.2	chymotrypsinogen B2	↓0.550	↓0.236	↓0.525	↓0.429		↓0.449
28	IPI00298497.3	Fibrinogen beta chain precursor	↓0.363	↓0.240	↓0.402	↓0.661	↓0.343	↓0.597
**29**	**IPI00856098.1**	**p180/ribosome receptor**	**↓0.555**	**↓0.242**	**↓0.614**	**↓0.437**	**↓0.247**	**↓0.394**
30	IPI00298547.3	PARK7 Protein DJ-1	↓0.192	↓0.249	↓0.324			
**31**	**IPI00550991.3**	**SERPINA3**	**↓0.242**	**↓0.250**	**↓0.154**	**↓0.329**	**↓0.131**	**↓0.234**
32	IPI00004457.3	AOC3 Membrane copper amine oxidase	↓0.214	↓0.261	↓0.429	↓0.242		↓0.256
33	IPI00218914.5	Retinal dehydrogenase 1	↓0.182	↓0.268	↓0.343	↓0.238	↓0.237	↓0.207
34	IPI00010133.3	Coronin-1A	↓0.353	↓0.281	↓0.586		↓0.510	↓0.167
35	IPI00015614.3	PRSS3 Isoform A of Trypsin-3 precursor	↓0.273	↓0.288	↓0.143			↑2.014
...	...	...	...	...	...	...	...	...
190	IPI00646304.4	PPIB peptidylprolyl isomerase B precursor	↑2.489	↑4.325	↑1.820	↑2.148		↑2.377
191	IPI00744692.1	TALDO1 Transaldolase	↑2.312	↑4.365		↑1.570		↑3.597
192	IPI00414283.5	fibronectin 1 isoform 4 preproprotein	↑1.959	↑4.365		↑2.228	↑2.466	
193	IPI00398778.3	PLEC1 plectin 1 isoform 10	↑1.871	↑4.406		↑10.765		↑7.586
**194**	**IPI00815665.1**	**PRSS1 protein**	**↑4.966**	**↑4.446**	**↑7.253**	**↑15.276**	**↑4.966**	**↑3.076**
195	IPI00218918.5	Annexin A1		↑4.487		↑2.831		↑3.631
196	IPI00021766.5	RTN4 Isoform 1 of Reticulon-4	↑3.221	↑4.529	↑3.253	↑20.512	↑4.328	↑16.596
197	IPI00215911.3	APEX1 DNA-(apurinic or apyrimidinic site) lyase		↑4.656	↑2.051	↑6.252	↑2.754	↑2.270
198	IPI00607708.3	LDHA Isoform 2 of L-lactate dehydrogenase A chain	↑6.918	↑4.656		↑12.134		↑10.375
199	IPI00796333.1	ALDOA 45 kDa protein	↑6.026	↑4.742		↑2.786	↑1.939	↑5.861
200	IPI00025512.2	Heat shock protein beta-1	↑2.208	↑4.786		↑4.831		↑4.487
201	IPI00186711.3	PLEC1 plectin 1 isoform 6	↑3.515	↑4.786	↑2.443	↑4.446	↑2.270	↑1.959
202	IPI00021827.3	DEFA3 Neutrophil defensin 3 precursor		↑4.831	↑2.512	↑2.512	↑3.212	↑1.923
...	...	...	...	...	...	...	...	...
216	IPI00218733.6	Superoxide dismutase	↑4.462	↑6.546		↑5.754		↑4.656
217	IPI00219038.9	H3F3B Histone H3.3	↑1.837	↑6.607		↑3.597		
218	IPI00000105.4	Major vault protein	↑4.128	↑6.855	↑2.629	↑4.699		↑4.207
219	IPI00465431.7	LGALS3 Galectin-3	↑7.112	↑6.918	↑2.466			↑2.805
**220**	**IPI00414676.6**	**Heat shock protein HSP 90**	**↑3.664**	**↑7.379**	**↑3.311**	**↑40.179**	**↑18.030**	**↑2.228**
221	IPI00019502.3	Myosin-9	↑4.525	↑7.586	↑3.342	↑7.112	↑3.133	↑2.270
222	IPI00887678.1	LOC654188 similar to peptidylprolyl isomerase A-like	↑4.920	↑7.656		↑12.134	↑1.770	↑6.855
223	IPI00178926.2	immunoglobulin J chain	↑2.512	↑7.727	↑1.629	↑40.551	↑8.551	↑4.742
**224**	**IPI00871843.1**	**TGM2**	**↑7.112**	**↑7.870**	**↑3.631**	**↑3.736**	**↑3.470**	**↑2.168**
225	IPI00289862.3	Secernin-1	↑1.959	↑7.870	↑1.660	↑2.148		↑4.742
226	IPI00018219.1	TGFBI Transforming growth factor-beta-induced protein ig-h3 precursor	↑1.542	↑8.241		↑17.701		↑29.923
227	IPI00027230.3	HSP90B1 Endoplasmin precursor	↑4.742	↑8.395	↑1.995	↑2.512		↑4.207
228	IPI00027463.1	Protein S100-A6	↑3.076	↑8.395	↑2.911	↑2.729		↑2.884
229	IPI00450768.7	type I cytoskeletal 17	↑2.228	↑8.710	↑2.333	↑3.908		↑3.733
230	IPI00449920.1	IGHV3OR16-13 cDNA FLJ90170 fis	↑2.089	↑9.120	↑2.014	↑4.365		↑4.529
231	IPI00293276.10	LOC284889; MIF Macrophage migration inhibitory factor	↑28.314	↑9.376	↑4.831	↑2.355		↑1.941
...	...	...	...	...	...	...	...	...
235	IPI00028030.3	COMP Cartilage oligomeric matrix protein precursor	↑1.660	↑12.023	↑3.192	↑8.930	↑55.463	↑3.767
236	IPI00002745.1	Cathepsin Z precursor	↑8.551	↑12.706	↑6.546		↑4.241	↑1.941
237	IPI00853163.1	TYMP 46 kDa protein	↑5.200	↑13.677		↑2.630		
238	IPI00329573.9	COL12A1 Isoform 1 of Collagen alpha-1(XII) chain precursor	↑4.267	↑14.997	↑4.366	↑19.055	↑5.620	↑13.804
239	IPI00022892.2	Thy-1 membrane glycoprotein precursor		↑16.749	↑9.290	↑1.614		↑1.803
240	IPI00009867.3	type II cytoskeletal 5	↑7.047	↑17.865		↑2.535		
241	IPI00642455.2	Thrombospondin 2		↑25.586	↑1.500	↑62.517	↑3.664	↑17.061
242	IPI00399007.5	IGHG2 Putative uncharacterized protein DKFZp686I04196 (Fragment)	↑33.113	↑17.701	↑34.995	↑18.707		
243	IPI00410241.2	POSTN Periostin, osteoblast specific factor	↑11.376	↑37.325		↑3.281		↑8.096

No, protein number; Accession^#^, protein IPI number in the database; NGM, normal gastric mucosa; AH, atypical hyperplasia; GPDAC, poorly differentiated gastric adenocarcinoma; LMGAC, lymph node metastasis adenocarcinoma; AH *vs*. NGM, GPDAC *vs*. NGM, LMGAC *vs*. NGM, GPDAC *vs*. AH, LMGAC *vs*. AH, and LMGAC *vs.* GPDAC represent the ratios of protein expression between the two indicated tissues.

**Table 2:** PRSS1 expression in gastric carcinoma

Protein	n	Score		
		Negative (-)	Positive (+-++)	Strongly positive (+++)
**PRSS1**				
NGM	34	30	4	0
AH	26	20	5	1
GWDAC	5	2	3	0
GMDAC	17	6	5	6
GPDAC	59	15	20	24
LMGAC	55	10	18	27

NGM, normal gastric mucosa; AH, atypical hyperplasia; GC, gastric carcinoma; GWDAC, well differentiated gastric adenocarcinoma; GMDAC, moderately differentiated gastric adenocarcinoma; GPDAC, poorly differentiated gastric adenocarcinoma; LMGAC, lymph node metastasis adenocarcinoma.

**Table 3:** Association of PRSS1 expression with demographic and clinical characteristics of patients with gastric carcinoma

Group	Cases (n)	Positive rate of PRSS1 (%)
**Sex**		
Male	56	71.43
Female	25	68
**NGM**	34	13.33
**AH**	26	23.08
**GC**		
GWDAC	5	60
GMDAC	17	64.71
GPDAC	59	74.58*
**Tumor size**		
>3.0 cm	49	65.31
≤3.0 cm	32	81.25*
**Lymph node metastasis**		
No	26	18.18
Yes	55	81.82**
**TNM staging**		
I-II	32	56.25
III-IV	49	81.62**

NGM, normal gastric mucosa; AH, atypical hyperplasia; GC, gastric carcinoma; GWDAC, well differentiated gastric adenocarcinoma; GMDAC, moderately differentiated gastric adenocarcinoma; GPDAC, poorly differentiated gastric adenocarcinoma; LMGAC, lymph node metastasis adenocarcinoma; *P<0.05, **P <0*.*01.

**Table 4:** PAR-2 expression in gastric carcinoma

Groups	Cases (n)	PAR-2 (n)				PAR-2 positive rate (%)
		Negative (-)	Weakly positive (+)	Moderately positive (++)	Strongly positive (+++)	
NGM	59	46	6	5	2	22.03
GWDAC	8	2	1	3	2	75.00*
GMDAC	29	3	4	5	17	89.66*
GPDAC	104	6	13	27	58	94.23*
LMGAC	53	3	4	12	34	94.34*

NGM, normal gastric mucosa; GWDAC, well-differentiated gastric adenocarcinoma; GMDAC, moderately differentiated gastric adenocarcinoma; GPDAC, poorly differentiated gastric adenocarcinoma; LMGAC, lymph node metastasis adenocarcinoma. *Compared with normal gastric mucosa, *P*=0.0418.

**Table 5:** Association between PAR-2 and clinicopathological characteristics in gastric carcinoma

Variables	Cases (n)	PAR-2 positive rate (%)	*P* value
**Sex**			*P*=0.7461
Male	90	91.11	
Female	51	94.12	
**Ages**			*P*=1.0000
≤60	58	93.10	
>60	83	91.57	
**Tumor differentiation**			*P*=0.1568
Well/moderately differentiated	37	86.48	
Poorly differentiated	104	94.23	
**TNM staging**			*P*=0.0001
I-II	29	72.41	
III-IV	112	97.32	
**Lymph node metastasis**			*P*=0.0122
No	30	80.00	
Yes	111	95.50	

GC, gastric carcinoma. *P<0.05, statistically significant.

## Abbreviations

GC: gastric carcinoma
LCM: laser capture microdissection
PRSS1: serine protease 1
iTRAQ: isobaric tags for relative and absolute quantitation
Q-TOF MS/MS: quadrupole time-of-flight tandem mass spectrometry
PAR-2: protease-activated receptor-2
NGM: normal gastric mucosa
AH: atypical hyperplasia
GPDAC: poorly differentiated gastric adenocarcinoma
LMGAC: lymph node metastasis adenocarcinoma
IHC: immunohistochemistry
ICC: immunocytochemistry
NC: negative control
MAPK: mitogen-activated protein kinase
PRSS1-WT: wild-type PRSS1
PCNA: proliferating cell nuclear antigen
miR-PRSS1: pcDNA6.2TM-GW/EmGFP-miR-PRSS1
PRSS1-NC: negative control plasmid pcDNA6.2TM-GW/EmGFP-miR
FSLLRY-NH2: PAR-inhibitor
SLIGKV-NH2: PAR-2 agonist
