# Supplementary material for: Silencing PRSS1 suppresses the growth and proliferation of gastric carcinoma cells via the ERK pathway
Source: Int J Biol Sci. 2021 Mar 1;17(4):957–71. doi: 10.7150/ijbs.52591 (PMC8040304; doi:10.7150/ijbs.52591)
Supplement: Supplementary file 1 — Supplementary tables. [file ijbsv17p0957s1.pdf]

Supplementary table 1. LCM purified poorly differentiated gastric adenocarcinoma

Cases (n=20)	Sex	Ages	Site of gastric cancer	Stage	Lymph node metastasis
1	male	41	gastric body	II	Yes
2	female	63	gastric antrum	IV	Yes
3	female	57	gastric fundus	IV	Yes
4	male	48	gastric antrum	II	Yes
5	male	58	gastric body	II	Yes
6	male	60	gastric body	II	Yes
7	male	77	gastric fundus	II	Yes
8	female	47	gastric body	III	Yes
9	male	69	gastric body	II	Yes
10	female	49	gastric antrum	II	Yes
11	female	67	gastric body	II	Yes
12	male	53	gastric fundus	II	Yes
13	male	54	gastric fundus	IV	Yes
14	male	70	gastric antrum	II	Yes
15	male	61	gastric antrum	II	Yes
16	male	50	gastric body	II	Yes
17	female	61	gastric fundus	III	Yes
18	male	71	gastric body	II	Yes
19	female	56	gastric body	II	Yes
20	female	38	gastric body	III	Yes
